# Effects of post‐hepatic portoenterostomy adjuvant therapy on liver transplantation in children with biliary atresia: A systematic review

**DOI:** 10.1002/jpr3.70214

**Published:** 2026-07-13

**Authors:** Bianca Ferraz de Almeida Silva, Alice Palmeira Nascimento Cerqueira, Breno Oliveira Marques, Gabriela Garcia de Carvalho Laguna, Gabrielle dos Santos Aguiar, Luana Leal Gonzaga, Raquel Teles de Lacerda, Natalia Oliveira e Silva

**Affiliations:** ^1^ Multidisciplinary Health Institute Federal University of Bahia Vitória da Conquista Bahia Brazil; ^2^ Federal University of Southern Bahia Bahia Brazil; ^3^ Pediatrics and Applied Pediatric Sciences, Federal University of Bahia Bahia Brazil

**Keywords:** antibiotics, corticosteroids, native liver survival

## Abstract

**Objectives:**

Biliary atresia (BA) is a cholangiopathy characterized by obstruction of the intrahepatic and extrahepatic bile ducts. Hepatic portoenterostomy (HPE) is the primary palliative treatment and there is still an urgent need to improve post‐HPE management. This study aims to identify post‐HPE adjuvant therapies associated with reducing or delaying the need for liver transplantation in children with BA.

**Methods:**

This systematic review was registered on the PROSPERO platform (ID: CRD42024553671). Studies were extracted from the following databases: PubMed, Web of Science, Lilacs, and Scielo. Original articles published between 2003 and 2025 in English, Portuguese, and Spanish were included, while studies involving adults, animals, or unrelated topics were excluded.

**Results:**

A total of 870 studies were identified, and after applying eligibility criteria, 16 articles were selected. The studies indicated that corticosteroids alone as adjuvant therapy were not associated with improved native liver survival. However, observational studies indicated that corticosteroid therapy combined with antibiotics, anticholestatics, and immunoglobulin may be associated with improvements in jaundice, although only two studies reported increased liver survival. Rectal budesonide demonstrated promising short and long‐term results in children with nonsyndromic BA.

**Conclusions:**

The use of adjuvant therapy after HPE was associated with improvements in jaundice and cholangitis and, in some studies, with increased liver survival. However, the findings are conflicting and heterogeneous. Further research is needed, with standardized therapeutic approaches to enable more comprehensive analyses.

## Abstract (highlights)

**What is Known:**

- There is currently no standardized adjuvant treatment following hepatic portoenterostomy.
- Postoperative outcomes may involve severe complications, including persistent cholestasis and even death.

**What is New:**

- Intravenous immunoglobulin (IVIg) has shown mixed results in studies but demonstrated positive outcomes in the subgroup of children with refractory post‐HPE cholangitis.
- Rectal budesonide is an emerging and promising therapy for nonsyndromic BA, underscoring the importance of conducting multicenter clinical trials to evaluate its efficacy.
- Standardization in the use of corticosteroids and IVIg is necessary to allow more consistent evaluations.

## 1 INTRODUCTION

Biliary atresia (BA) is a rare, progressive fibroinflammatory disease classified among the cholangiopathies and characterized by obstruction of the intrahepatic and extrahepatic bile ducts.^1^, ^2^ Its etiology, however, remains unknown. BA manifests during the neonatal period, is the most common cause of end‐stage liver failure, and is the leading indication for liver transplantation in children.^3^ If left untreated, BA progresses to cirrhosis, leading to death by the second year of life.^4^ Its prevalence is estimated at 5 to 10 per 100,000 live births, varying by geographic region,^5^ with the highest prevalence observed in East Asian countries, particularly Taiwan.^6^

The main clinical presentation of BA is persistent icteric cholestasis.^7^ Hepatic portoenterostomy (HPE) is the primary palliative treatment, aiming to restore bile flow.^8^ Currently, HPE success is defined as achieving a total bilirubin (TB) level below 2 mg/dL within the first 3 months after drainage.^9^ There remains an urgent need to optimize post‐HPE management. The objective is to achieve even more favorable outcomes, including the prevention of complications, further delay of definitive treatment, and improved patient survival.^3^, ^7^

Post‐HPE adjuvant treatments are designed to alleviate cholestasis, prevent inflammation and cholangitis, and ensure adequate nutrition.^7^ Therefore, this study aims to identify post‐HPE adjuvant therapies associated with reducing or delaying liver transplantation in children with BA.

## 2 METHODS

### 2.1 Ethics statement

The authors declare no conflicts of interest or sources of funding. This study did not require approval by an ethics committee.

### 2.2 Study design and research question

This article is a systematic review registered in the PROSPERO platform (ID: CRD42024553671), aiming to support evidence‐based decision‐making,^10^ and conducted in accordance with Preferred Reporting Items for Systematic Reviews and Meta‐Analyses (PRISMA) guidelines, as documented in Table S1.^11^ The study was based on the following question: “Is the use of post‐HPE adjuvant therapy (I) associated with the reduction or delay of liver transplantation (O) in children with biliary atresia (P)?” following the PICO strategy.^11^

### 2.3 Search strategy

Studies were retrieved from PubMed, Web of Science, Lilacs, and Scielo. The following descriptors were used: (Biliary Atresia); (Glucocorticoids); (Transplantation); (Treatment); (Hepatic Portoenterostomy). The search strategy applied in each database was: (Hepatic Portoenterostomy) AND (Biliary Atresia) AND (Treatment) AND (Transplantation); and (Hepatic Portoenterostomy) AND (Biliary Atresia) AND (Glucocorticoids) AND (Transplantation).

### 2.4 Inclusion and exclusion criteria

Original articles published between 2003 and 2025 in English, Portuguese, or Spanish were included if they addressed post‐HPE adjuvant therapy in children with BA. Exclusion criteria were duplicate studies, studies conducted in adults or animals, case reports, and studies that did not address the research question.

### 2.5 Study selection and data extraction

Study selection was performed by two blinded and independent reviewers (B.F.A.S. and G.G.C.L.) following the inclusion and exclusion criteria, with screening conducted using the Rayyan platform.^12^ Initial screening was based on abstracts and titles, followed by full‐text review of eligible studies. Discrepancies were resolved by consensus. Data from the selected studies were then extracted and organized using Microsoft Excel. Variables collected and analyzed included author, study design, year and location, sample size, intervention, therapeutic dose, frequency and duration of intervention, and main outcomes. Data extraction was performed by three independent reviewers.

### 2.6 Quality assessment

Quality assessment of eligible studies was performed using the Jadad scale^13^, ^14^ for randomized clinical trials, which evaluates three aspects of study design through five binary questions (“Yes” = 1; “No” = 0) regarding randomization, blinding, and withdrawals/losses. For observational studies, including cohort and case‐control studies, the Newcastle‐Ottawa Scale (NOS) was used.^15^ Stars were assigned according to the scale: selection (maximum 4 stars), comparability (maximum 2 stars), and outcome/exposure (maximum 3 stars). Study quality was classified as “good” if scoring 3–4 stars in selection, 1–2 in comparability, and 2–3 in outcome/exposure; “fair” if scoring 2 stars in selection, 1–2 in comparability, and 2–3 in outcome/exposure; and “poor” if scoring 0–1 star in selection, 0 in comparability, and 0–1 in outcome/exposure. Each article was evaluated by three independent reviewers.

### 2.7 Statistical analysis

Due to methodological heterogeneity and the small number of eligible studies, meta‐analysis was not performed, and a descriptive synthesis of the results was conducted.

## 3 RESULTS

A total of 871 records were identified in the databases investigated, of which 634, published between 2003 and 2025, were screened. After applying the eligibility criteria, 16 articles were included for analysis, as shown in the selection flowchart in Figure 1. Among the included studies, 11 were observational cohorts, 5 were clinical trials, and notably, only one was a double‐blind, placebo‐controlled trial.^16^ The quality assessment of each study and their individual characteristics are presented in Tables 1 and 2, respectively. The PRISMA checklist is provided in Table S1. Table S2 provides a description of all drugs used in the included studies and Figure 2 summarizes the main treatments studied and the new therapies under investigation.

**Figure 1:**
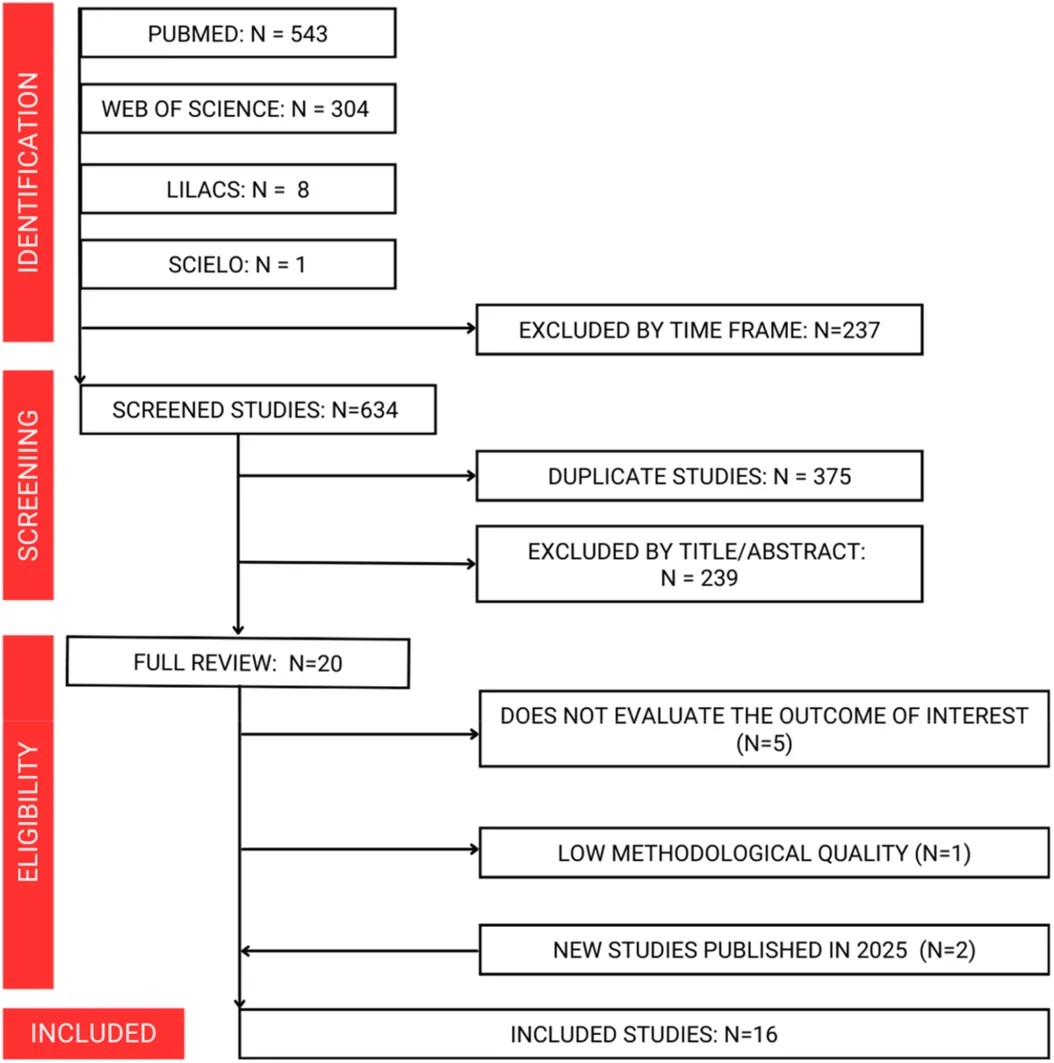
Study selection flowchart. Prepared by the authors (2025).

**Table 1:** Quality of studies based on the NOS for cohort and case control studies and the Jadad Scale for randomized clinical trials.

Study	Study design	Scale	Classification
Abanemai, 2023, Saudi Arabia	Retrospective cohort	NOS	Good
Escobar, 2006, USA	Retrospective cohort	NOS	Good
Takeda, 2023, Japan	Retrospective cohort	NOS	Reasonable
Susuki, 2010, Japan	Retrospective cohort	NOS	Reasonable
Meyers, 2003, USA	Retrospective cohort	NOS	Good
Pandurangi, 2022, USA	Retrospective cohort	NOS	Good
Ramos‐Gonzalez, 2019, USA	Retrospective cohort	NOS	Good
Davenport, 2007, United Kingdom	Randomized clinical trial	Jadad scale	High quality
Bezerra, 2014, USA	Randomized clinical trial	Jadad scale	High quality
Pietrobattista, 2020, Italy	Retrospective cohort	NOS	Good
Bu, 2003, Taiwan	Randomized clinical trial	Jadad scale	High quality
Stringer, 2007, United Kingdom	Prospective cohort	NOS	Reasonable
Li, 2018, China	Retrospective cohort	NOS	Good
Mack, 2020, USA	Phase I/IIa, multicenter, single‐arm, open‐label clinical trial	NOS	Good
Harpavat, 2025, USA	Phase II, nonrandomized, open‐label	NOS	Good
Langreen, 2025, Germany	Cohort	NOS	Good
Davenport, 2013, United Kingdom	Randomized clinical trial	Jadad scale	Low quality

*Note*: Prepared by the authors (2025).

Abbreviations: NOS, Newcastle‐Ottawa Scale; USA, United States of America.

**Table 2:** Description of the investigated therapies and characterization of the studies with their main results.

Study	Intervention	Results
Bezerra, 2014, USA. Double‐blind Placebo‐controlled trial (NCT00294684)	140 participants, 70 from the steroid group. Methylprednisolone (4 mg/kg/day for 2 weeks) or prednisolone (2 mg/kg/day for 2 weeks), initiated within 72 h after HPE, followed by a tapering protocol for 9 weeks.	Steroid treatment did not increase the proportion of participants achieving the primary endpoint of serum TB level less than 1.5 mg/dL. Secondary outcomes were survival without liver transplantation and adverse events. The need for liver transplantation for participants treated with steroids was almost identical to that for those receiving placebo (58.7% vs. 59.4%). Patients treated with steroids experienced their first serious adverse events earlier than those receiving placebo.
Abanemai, 2023, Saudi Arabia. Retrospective cohort	204 participants, 143 undergoing HPE, 45 using steroids. IV methylprednisolone and/or oral prednisone in different protocols with the median duration was 30 days. IV antibiotics after HPE.	Steroid use was associated with significant improvement in jaundice (68% vs. 36.8%; *p* = 0.013, Odds Ratio 2.5) and with an improvement in the survival rate with native liver at 2 and 10 years (62.22% and 57.77% vs. 39.47% and 31.57%; *p* = 0.01).
Escobar, 2006, USA. Retrospective cohort	43 participants, 21 of which were steroid group. Prednisone and dexamethasone. Most common doses of 1.5; 2 and 2.5 mg. Prophylactic antibiotics (ampicillin, gentamicin and clindamycin) from 1 day to 1 month, choleretics (ursodeoxycholic acid and phenobarbital), vitamins A, D, E, and K.	Normal postoperative bilirubin achieved at 6 months in 76% of the steroid group (vs. 37% control group). Need for liver transplant in 19 patients, 37% of the steroid group (vs. 63% control group). Post‐HPE cholangitis in 25 patients, 12 in the steroid group (vs. 13 in the control group).
Takeda, 2023, Japan. Retrospective cohort	47 participants on steroids. IV prednisolone starting with 4 mg/kg/day, followed by 3 mg/kg, 2 mg/kg, 1 mg/kg, and 0.5 mg; the total dose of prednisolone per 15‐day cycle was 31.5 mg/kg. The cycles were restarted until the patient achieved clearance of jaundice and normalization of stool color.	Overall reduction of jaundice, TB up to 1.2 mg/dL, in 42 of 47 patients (89.4%).
Susuki, 2010, Japan. Retrospective cohort	53 participants undergoing corticosteroid. Hydrocortisone, 100 mg (1 dose) immediately after surgery, followed by IV administration of prednisolone, 4 mg/kg, from the second postoperative day, with a gradual reduction in the total daily dose by 4 mg every 3 days. Ursodeoxycholic acid and prophylactic antibiotic (second generation cephalosporin + aminoglycoside) until CRP normalizes	43 (81.1%) returned to normal TB, with time varying from 12 to 293 days (median of 45 days); 39 of the 53 patients (73.6%) remained alive without the need for liver transplantation. 11 underwent liver transplantation, of which 3 died.
Meyers, 2003, USA. Retrospective cohort	28 participants, 14 in the steroids group. IV methylprednisolone (tapering 10, 8, 6, 5, 4, 3, and 2 mg/kg/day) followed by 8–12 weeks of oral prednisone (2 mg/kg/day). Oral ursodeoxycholic acid indefinitely every 12 h. IV antibiotics for 8–12 weeks (piperacillin/tazobactam plus gentamicin or cefoperazone, followed by oral TMP/SMZ	79% in the steroid group and 21% in the standard therapy group had a conjugated bilirubin level less than 1.0 within 3–4 months after surgery (*p* < 0.001). Fewer patients in the steroid group (21% vs. 85%) required liver transplantation or died during the first year of life (*p* < 0.001).
Pandurangi, 2022, USA. Retrospective cohort	40 participants (20 intervention). All received IV Cefoxitin (3–4 days). Subsequent therapy was stratified by stool color and histology: standard cases received TMP/SMZ, vitamins, and ursodeoxycholic acid. High‐risk cases (<45 days with stool abnormalities or >45 days with inflammation) received 5‐day IV antibiotics and tapering Methylprednisolone, followed by oral Prednisolone and Amoxicillin‐Clavulanate.	TB 3 months after HPE was <2 mg/dL in 16 of 20 patients (80%) treated with the new protocol compared with 8 of 20 patients in the control group (40%, *p* = 0.0225). Survival with native liver at 24 months increased with the new protocol, but did not reach statistical significance.
Ramos‐Gonzalez, 2019, USA. Retrospective cohort	81 participants, 42 on steroids. A uniform steroid protocol was not used. Most patients started on a postoperative steroid dose of intravenous methylprednisolone 4 mg/kg/day.	The median follow‐up time was 5.7 years (IQR: 1–11.6). The 10‐year overall survival was 93% (95% CI: 84–97). Thirty‐six patients (44%) required a transplant at a mean time from HPE of 8.9 months (IQR: 5.2–19). The 10‐year transplant‐free survival was 36% (95% CI: 24–49). Steroid use was not associated with an improvement in overall survival or transplant‐free survival.
Davenport, 2007, United Kingdom. Randomized clinical trial (registration number not reported)	71 participants, 36 from the steroid group. Oral prednisolone (2 mg/kg/day from day 7 to day 21 followed by 1 mg/kg/day from Day 22 to Day 28). Oral ranitidine (during corticosteroid treatment). Routine immunizations were postponed for 1 month.	At 1 month, the mean bilirubin level was lower in the steroid group (66 vs. 92 mol/L, *p* = 0.06), but no difference was evident at 6 or 12 months. The need for transplantation at 6 (12% vs. 13%, P 0.99) and 12 months (26% vs. 35%, *p* = 0.47) was not significantly different.
Pietrobattista, 2020, Italy. Retrospective cohort	43 participants, 25 undergoing adjuvant therapy. Prophylactic antibiotic: ceftriaxone for 5 days, followed by amikacin for 2 days. Methylprednisolone 10 mg/kg/day for a total of 5 days. At this point, oral prednisolone 2 mg/kg/day was replaced with a 50% dose reduction every 15 days until discontinuation. Amoxicillin‐clavulanate and TMP for 12 months after surgery.	Clinical complications of liver disease were similar between the two groups. After 6 months of HPE, there was no significant difference between the two groups in terms of Pediatric End‐stage liver disease scores and achievement of TB ≤ 1.5 mg/dL. There was no benefit in the incidence of cholangitis or survival with native liver at 12 months.
Bu, 2003, Taiwan. Randomized clinical trial (registration number not reported)	37 participants, 19 undergoing the proposed adjuvant therapy. TMP/SMZ or neomycin orally for prophylaxis against recurrent cholangitis up to three years of age	There was no difference in cholangitis recurrence rates between the TMP/SMZ and neomycin groups; survival rates were higher in the TMP/SMZ and neomycin groups than in the control group. There were 1, 2, and 4 cases who received liver transplantation in the TMP/SMZ, neomycin, and control groups, respectively.
Stringer, 2007, United Kingdom. Prospective cohort	60 participants, 50 under the adjuvant therapy (group 1), 10 in control with Prednisolone (group 2). Oral ursodeoxycholic acid twice a day. Oral dexamethasone starting on the 5th day of postoperative. Phenobarbital and oral ranitidine while taking steroids. 5‐day course of IV amoxicillin and gentamicin followed by oral cephaladine for 4 weeks.	Overall, 42 of 60 babies cleared jaundice (76% adjuvant therapy group vs. 40% prednisolone or placebo group). There was no significant difference in overall survival between group 1 (*n* = 50, 92% survival) and group 2 (*n* = 10, 100% survival). Liver transplantation was necessary and performed in 25% of group 1 (12, two of whom died) and 70% of group 2 (7—all survived).
Li, 2018, China. Retrospective cohort	29 patients with intractable cholangitis (16 intervention, 13 control). IVIg (400 mg/kg/day for 3 days), IV meropenem, metronidazole, oral prednisone (5 mg/kg/day for 5 days, followed by 1‐week taper), and oral ursodeoxycholic acid (twice daily).	The IVIg group had a shorter duration of fever after treatment (*p* = 0.011) and length of hospital stay than the control group (*p* = 0.018). The outcomes were as follows (IVIg vs. control group): episode of recurrent cholangitis (68.7 vs. 92.3%), liver transplant (38.5 vs. 46.2%), survival with native liver (25 vs. 15.4%), and death (50 vs. 61.5%). Time to recurrent episode of cholangitis was statistically different between groups: (range 13–121 days in IVIg group vs. range 2–80 days in control group).
Mack, 2020, USA. Phase I/IIa, multicenter, single‐arm, open‐label clinical trial (NCT01854827)	29 participants with BA post‐HPE. 1 g/kg/dose of IVIg infused at 3–5 days, 30 days, and 60 days post‐HPE, and subjects followed for 360 days post‐HPE. Oral ursodeoxycholic acid for 360 days after HPE. TMP/SMZ for 180 days after HPE. High‐MCT containing infant formula, and vitamin supplementation.	Primary outcomes addressed the feasibility, acceptability, and safety of IVIg. Secondary outcome measures included efficacy (good bile drainage) and SNL. No severe adverse events were directly associated with IVIg. Compared to the placebo group, there was no significant increase in patients with TB < 1.5 mg/dL at 90, 180, or 360 days after HPE. Survival with the native liver in patients who received IVIg did not show a statistically significant benefit compared to the placebo group.
Langreen, 2025, Germany. Retrospective cohort	279 participants, 142 patients in the study group. Rectal foam budesonide 2 mg/dose administered on day 5 or 3 after HPE once a day for 3 months. Antibiotics for 10–14 days, oral antibiotics for 6 months, long‐term fat soluble vitamins and ursodeoxycholic acid.	Improvements in jaundice‐free native liver survival (jfNLS) were observed at 6 months (53% vs. 39%) and 2 years (45% vs. 22%), with sustained benefits at 5 years (40% vs. 23%) and 10 years (32% vs. 13%). These benefits were exclusive to patients with nonsyndromic BA. No serious adverse effects were observed.
Harpavat, 2025, USA. Phase II, nonrandomized, open‐label (NCT03499249)	12 participants compared with 24 historical control patients. IV NAC 150 mg/kg/day was administered continuously for 7 days, starting within the first 24 h following HPE. Ursodeoxycholic acid, TMP‐SMZ, fat‐soluble vitamin supplementation, and high‐MCT.	The primary endpoint was achieving good bile drainage, as defined by TSBA ≤ 10 μmol/L with native liver within the first 24 weeks following HPE. The secondary endpoints were anthropomorphic measurements and laboratory values. The endpoint of TSBA ≤ 10 micromol/L was not achieved. No significant differences were observed regarding markers of liver injury and function. No differences were observed in relation to liver transplantation and death.

*Note*: Prepared by the authors (2025).

Abbreviations: BA, biliary atresia; CI, confidence interval; CRP, C‐reactive protein; High‐MCT, high medium‐chain triglycerides; HPE, hepatic portoenterostomy; IQR, interquartile range; IV, Intravenous; IVIg, intravenous Immunoglobulin; jfNLS, Jaundice‐free native liver survival; MCT, medium‐chain triglycerides; NAC, N‐acetylcysteine; NOS, Newcastle‐Ottawa Scale; SNL, survival with native liver; TB, total bilirubin; TMP/SMZ, trimethoprim‐sulfamethoxazole; TSBA, total serum bile acids.

**Figure 2:**
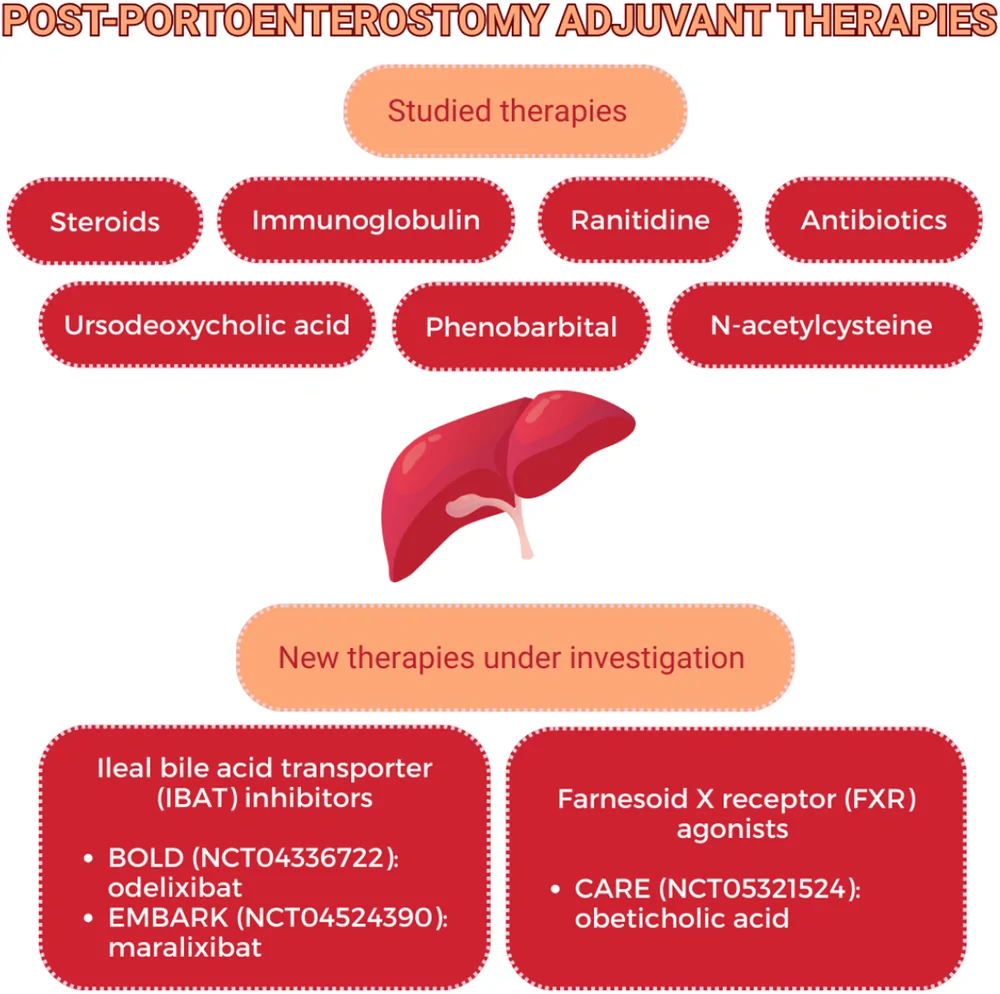
Post‐HPE adjuvant treatments. BA, Biliary atresia; BOLD, efficacy and safety of odevixibat in children with BA who have undergone a Kasai HPE; CARE, obeticholic acid in pediatric subjects with BA; EMBARK, evaluation of maralixibat in BA response post‐Kasai; HPE, hepatic portoenterostomy. Prepared by the authors (2025).

### 3.1 Jaundice clearance

A retrospective observational study from Saudi Arabia^17^ reported improvements in jaundice control with intravenous methylprednisolone and oral prednisone (*p* = 0.01). Improvement was also observed with prednisone therapy guided by stool color monitoring in two retrospective studies.^18^, ^19^ The use of dexamethasone and other corticosteroids for periods ranging from 2 to 6 weeks, combined with antibiotics and anticholestatic agents, effectively reduced total serum bilirubin (76% of patients in the steroid group vs. 37% in the control group; *p* = 0.01).^18^ Administration of a single dose of hydrocortisone followed by a tapered prednisolone regimen was likewise associated with improved bilirubin levels in a retrospective cohort.^20^ Another cohort reported that a protocol of 7 days of intravenous methylprednisolone followed by 8–12 weeks of oral prednisone resulted in bilirubin levels below 1 mg/dL in most patients in the corticosteroid group (*p* < 0.001).^21^ This study protocol included intravenous antibiotics and oral ursodeoxycholic acid. A prospective observational study indicated that dexamethasone combined with ursodeoxycholic acid improved bilirubin levels.^22^

Most protocols combined corticosteroid therapy with intravenous antibiotics and anticholestatic agents, particularly ursodeoxycholic acid. The use of beta‐lactam antibiotics and aminoglycosides alongside corticosteroids and ursodeoxycholic acid was associated with reduced TB levels and improved jaundice.^18^, ^19^, ^20^, ^21^, ^23^ A retrospective cohort protocol combining intravenous methylprednisolone and oral prednisolone with beta‐lactam antibiotics showed that TB 3 months post‐HPE was <2 mg/dL in 80% of patients receiving the new protocol compared to 40% in the control group (*p* = 0.0225).^23^ A clinical trial indicated no significant increase in the number of patients achieving TB <1.5 mg/dL at 90, 180, or 360 days post‐HPE with the use of Intravenous immunoglobulin (IVIg).^24^ Similarly, conflicting results were reported for the combination of methylprednisolone, prednisolone, ceftriaxone, amoxicillin‐clavulanate, and trimethoprim in a retrospective cohort. There was no association with TB levels below 1.5 mg/dL at 6 months.^25^

### 3.2 Transplant‐free survival

Most studies reported no statistically significant difference in liver survival when comparing adjuvant therapies with placebo.^16^, ^23^, ^25^, ^26^, ^27^ A cohort conducted in the United States indicated that corticosteroid use was not associated with improved transplant‐free survival at 10 years (33% vs. 38%, *p* = 0.690).^26^ The combination of methylprednisolone, prednisolone, ceftriaxone, amoxicillin‐clavulanate, and trimethoprim did not improve liver survival in a retrospective study.^25^ Regarding other therapies, in a randomized trial survival rates were higher in the trimethoprim‐sulfamethoxazole (TMP/SMZ) and neomycin groups than in controls, but only the comparison between the neomycin and control groups reached statistical significance.^28^ Neomycin and TMP/SMZ were associated with lower cholangitis rates compared to placebo.^28^

However, retrospective cohorts found lower liver transplant rates in children treated with corticosteroids, with native liver survival ranging from 2 to 10 years (*p* = 0.01).^17^, ^18^ An observational study reported that corticosteroid therapy combined with gentamicin, clindamycin, ampicillin, choleretics, phenobarbital, and fat‐soluble vitamins was associated with decreased need for transplantation (37% in the steroid group vs. 63% in the control group).^18^ Stringer and collaborators reported in a prospective cohort that dexamethasone combined with ursodeoxycholic acid reduced the need for transplantation compared to oral prednisolone therapy (25% vs. 70%, respectively).^22^

IVIg has been considered an alternative for intractable post‐HPE cholangitis in a retrospective cohort, showing improved liver survival in the IVIg group compared to the conservative treatment group (25% vs. 15.4%, respectively).^29^ However, native liver survival in the IVIg group was not significantly higher compared to controls in a clinical trial conducted between 2013 and 2016.^24^ A historical cohort in Germany including 279 patients demonstrated that rectal budesonide was associated with improved jaundice‐free native liver survival in patients with nonsyndromic BA at 6 months, 2 years, 5 years, and 10 years.^30^ The second phase of a clinical trial with N‐acetylcysteine (NAC) showed no improvement in laboratory parameters or long‐term outcomes, with no significant differences in transplantation or mortality.^31^ One serious adverse event was associated with NAC. The absence of positive outcomes led to discontinuation of progression to a phase III trial.

## 4 DISCUSSION

The data presented in this review reveal considerable divergence among therapeutic modalities and intervention protocols in patients, with variations in administration methods, dosages, treatment durations, and outcome descriptions. These issues are discussed throughout the text; however, definitive conclusions remain elusive due to methodological variability and conflicting results.

The success of HPE is limited by profound hepatotoxic factors already present at the time of intervention, with studies reporting that only 20%–40% of procedures independently lead to long‐term beneficial effects.^32^ These results remain inconsistent, with some studies reporting benefits in about 50% of children post‐HPE.^3^, ^33^, ^34^ Despite advancements in surgical techniques, achieving positive outcomes remains a challenge. A comparison between laparoscopic portoenterostomy and conventional surgery indicated that laparoscopy was more frequently associated with unfavorable outcomes, with conventional surgery still considered the preferred approach.^35^, ^36^, ^37^

To improve outcomes and the postoperative course in these patients, adjuvant therapies have been employed; however, their use lacks standardization and robust evidence to support them with greater certainty. The studies included in this review evaluated the use of adjuvant therapy post‐HPE in children with BA. Different drug classes, such as glucocorticoids, antibiotics, and choleretics, were used, with varying drugs, dosages, and regimens. Currently, no standardized protocol exists regarding therapeutic dosing in children with BA post‐HPE.

The use of glucocorticoids has been justified by the potential role of inflammation in the pathogenesis of BA, involving infiltrating CD4+ lymphocytes and natural killer (NK) cells, with a predominance of T helper 1 (Th1) and T helper 17 (Th17) cells, in addition to elevated plasma concentrations of molecules such as soluble intercellular adhesion molecule, soluble vascular cell adhesion molecule, and cytokines including interleukin‐2 (IL‐2), IL‐4, 18‐IL, tumor necrosis factor, and interferon. These markers are present not only at the time of HPE but also up to 6 months postoperatively, resolving after cholestasis recovery.^34^, ^38^, ^39^ Among the studies analyzed, interventions with glucocorticoid therapy alone or in combination showed varying results with divergent benefits.

Although some studies adopted similar interventions, no standardized approach has been consistently followed. Certain studies reported decreased need for liver transplantation in groups receiving corticosteroid therapy compared to controls, as well as significant improvements in jaundice and increased native liver survival rates at 2 and 10 years (*p* = 0.01) with the use of methylprednisolone and oral prednisone.^17^, ^19^ Conversely, other studies using intravenous methylprednisolone alone or combined with oral prednisone, including two randomized clinical trials, did not demonstrate superior benefits compared to patients who did not receive post‐HPE corticosteroid therapy. In these cases, transplant requirements and native liver survival did not differ significantly from the control group (*p* = 0.690).^16^, ^26^, ^27^

Antibiotics have been considered alongside corticosteroids to improve outcomes, primarily by reducing cholangitis. However, their prophylactic use lacks strong evidence regarding efficacy and safety.^34^, ^40^ Our review identified studies employing antibiotics either alone or in combination with steroids, with varied results. Prednisone, dexamethasone, and intravenous methylprednisolone were combined with different antibiotics. Prednisone and dexamethasone, administered alongside prophylactic antibiotics and choleretics, reduced the need for liver transplantation in a retrospective study.^19^ However, a randomized trial did not demonstrate a reduction in the need for transplantation with the use of prednisolone.^27^ Intravenous methylprednisolone followed by prednisone with prolonged intravenous antibiotics and choleretics also showed benefits.^21^ Similarly, hydrocortisone and prednisone with antibiotics and choleretics ^20^ and oral dexamethasone with ursodeoxycholic acid, phenobarbital, ranitidine, and antibiotics^22^ were associated with reduced transplantation rates.

A cohort study conducted in the United States of America^23^ proposed a new treatment protocol consisting of cefoxitin or piperacillin‐tazobactam and intravenous methylprednisolone, followed by corticosteroids and oral amoxicillin‐clavulanate. This regimen increased native liver survival in the intervention group, although the difference was not statistically significant. In contrast, a cohort study by Pietrobattista and colleagues^25^ did not show benefits from prophylactic ceftriaxone, amikacin, amoxicillin‐clavulanic acid, or trimethoprim combined with methylprednisolone as adjuvant therapy.

The use of prophylactic antibiotics was also associated in one study with a lower rate of liver transplantation.^28^ The regimen included TMP/SMZ or neomycin until the age of 3 years to prevent recurrent cholangitis. Patients receiving antibiotics had reduced incidence of cholangitis and a lower need for transplantation.

IVIg combined with a standard protocol (intravenous meropenem, metronidazole, oral prednisone, and ursodeoxycholic acid) reduced transplantation in children post‐HPE with intractable cholangitis.^29^ Its addition improved native liver survival compared to the standard protocol alone. IVIg has been associated with lower bilirubin levels, reduced vascular cell adhesion molecule‐1 (VCAM‐1) expression in the portal epithelium, and decreased cytokine production by T cells after high‐dose IgG therapy in a mouse model.^34^, ^41^ However, one study found no significant differences between IVIg and placebo in the proportion of patients with TB < 1.5 mg/dL, underscoring the need for further investigation.^24^ Methodological differences, including corticosteroid use and sample sizes, must be considered when comparing results. These inconsistencies highlight the need for studies to clarify IVIg's role in BA and optimize combination protocols, dosages, treatment duration, and other variables.

New therapies have been explored in an attempt to improve clinical outcomes. NAC, a precursor of glutathione with antioxidant properties, is essential for hepatocyte and cholangiocyte function.^42^, ^43^ Although animal models have demonstrated reduced liver injury in cholestasis and BA, no clinical data support its efficacy in humans.^44^ The trial conducted by Harpavat and colleagues further reinforces this lack of benefit; however, its small sample size (*n* = 12) and short treatment duration limit conclusions, highlighting the need for additional studies.^31^ Rectal budesonide has shown favorable results in patients with nonsyndromic BA. This therapy appears promising and has not been associated with adverse effects comparable to those of systemic corticosteroids.^30^ New clinical trials with ileal bile acid transporter (IBAT) inhibitors are ongoing. The BOLD study is evaluating daily odevixibat (NCT04336722) over 104 weeks, focusing on long‐term native liver survival and symptom management.^45^ The EMBARK trial (NCT04524390) is assessing maralixibat (*n* = 40) compared with placebo (*n* = 35). Preliminary results indicate no significant differences in serum TB levels, bile acid concentrations, or long‐term outcomes such as transplantation and mortality.^46^ The CARE study (NCT05321524) is underway to assess the safety, tolerability, and pharmacokinetics of obeticholic acid in patients aged 2–17 years with BA post‐HPE.^47^ Additionally, other drugs such as fibrates are being investigated in cholangiopathies and may represent potential alternatives for trials in BA patients.^48^

Considering these findings, further studies are needed to standardize dosages and assess factors such as age, nutritional status, disease severity, and biochemical markers for early prognostic evaluation. Multidisciplinary approaches should aim not only to reduce liver transplantation rates but also to preserve quality of life. Long‐term follow‐up is crucial to monitor adverse effects of adjuvant therapies, including the impact of prolonged antibiotic use on the intestinal microbiota.

The main limitations of this review are the limited number of available studies and significant methodological variability, largely due to the diversity of therapeutic regimens. Notably, some studies lacked details on steroid types and regimens,^17^, ^21^ used dual corticosteroid therapy,^27^ or varied steroid drugs.^21^, ^23^ Additionally, certain studies on corticosteroids did not specify concomitant use of antibiotics and choleretics.^16^, ^17^, ^18^, ^19^, ^20^, ^21^, ^22^, ^23^, ^25^, ^29^

## 5 CONCLUSION

The studies included in this review demonstrated mixed outcomes regarding reductions in liver transplantation rates with adjuvant therapy in children with BA post‐HPE. Divergence was observed in the use of corticosteroids, whether alone or in combination with other drugs. Antibiotics also indicated conflicting results, although most studies reported favorable outcomes. IVIg has yielded inconsistent findings but appears promising in children with intractable cholangitis, warranting further investigation. Among emerging therapies, rectal budesonide has shown positive results in cases of nonsyndromic BA in an observational study.

Further studies are needed to support recommendations for adjuvant therapy post‐HPE. Large, multicenter trials with standardized dosages and treatment protocols are essential for more effective and safe evaluation of adjuvant drugs on liver survival.

## CONFLICT OF INTEREST STATEMENT

The authors declare no conflicts of interest.

## Supporting information

Supplemental Table 1: Preferred Reporting Items for Systematic Reviews and Meta‐Analyses (PRISMA) Checklist.

Supplemental Table 2: Description of all therapies employed in the included studies.
