# Supplementary material for: Effects of post‐hepatic portoenterostomy adjuvant therapy on liver transplantation in children with biliary atresia: A systematic review
Source: JPGN Rep. 2026 Jul 13;7(4):572–81. doi: 10.1002/jpr3.70214 (PMC13394905; doi:10.1002/jpr3.70214)
Supplement: Supplementary file 1 [file JPR3-7--s001.docx]

Supplemental Table 2 - Description of all therapies employed in the included studies.

| **Steroids** | **Antibiotics** | **Others** |
| --- | --- | --- |
| Dexamethasone  Hydrocortisone  Methylprednisolone  Prednisolone  Prednisone  Rectal budesonide | Amoxicillin-clavulanate  Ampicillin  Cefoperazone  Ceftriaxone  Cefoxitin  Cephaladine  Clindamycin  Gentamicin  Meropenem  Metronidazole  Neomycin  Piperacillin/tazobactam  Sulfamethoxazole/trimethoprim  Trimethoprim | N-acetylcysteine  Intravenous Immunoglobulin (IVIg)  Phenobarbital  Ranitidine  Ursodeoxycholic acid  Vitamins A, D, E and K. |

Prepared by the authors (2025). Caption: Intravenous Immunoglobulin (IVIg).
